# The Relation between Asthma Control and Quality of Life in Children

**DOI:** 10.1155/2018/6517329

**Published:** 2018-07-03

**Authors:** M. Banjari, Y. Kano, S. Almadani, A. Basakran, M. Al-Hindi, T. Alahmadi

**Affiliations:** ^1^King Abdulaziz University, Jeddah, Saudi Arabia; ^2^University of Jeddah, Jeddah, Saudi Arabia; ^3^Department of Pediatrics, King Saud Bin Abdul Aziz University for Health Sciences, King Abdullah International Medical Research Centre, Jeddah, Saudi Arabia; ^4^Department of Paediatrics, King Abdulaziz University, Jeddah, Saudi Arabia

## Abstract

**Background:**

Asthma is a common chronic illness worldwide. Asthmatic children are forced to alter their way of living to avoid its complications or exacerbations, which negatively affects their psychological and social well-being. High prevalence of behavioral and emotional difficulties was noticed among children with asthma.

**Methods:**

Cross-sectional study that was conducted over 8 months involving asthmatic children within the ages of 7-17 years presenting to two governmental hospitals in Jeddah, Saudi Arabia. Three questionnaires were used: asthma control test, the strengths and difficulties questionnaire, and the pediatrics asthma quality of life questionnaire. Using SPSS, Pearson's chi-square and independent sample t-tests were used to find associations.

**Results:**

Among the 106 respondents, 84% of the sample had poor asthma control. Significantly poorer quality of life was observed in children with uncontrolled asthma (p = <0.001). Children with controlled and uncontrolled asthma were equally affected psychosocially with no relation between asthma control and their psychosocial well-being (p = 0.58).

**Conclusion:**

The majority of asthmatic children were uncontrolled with poor quality of life. This study recommends that the psychosocial well-being should be assessed during clinic visits for a better holistic approach and effective improvement of outcome. Further researches are needed to study the psychological effect of asthma.

## 1. Introduction

Asthma is one of the common chronic inflammatory diseases that primarily affects the airways. It has been estimated that around 300 million individuals in the world currently have asthma [1]. Asthma prevalence varies among different cities in Saudi Arabia. For example, a recent study was done in Al-Medina, Saudi Arabia, which revealed that the prevalence of primary school children diagnosed with asthma was 15.5% [2]. While another study done on schoolchildren in Najran during 2016 revealed that the prevalence rate of asthma was 27.4% [3].

The extent of asthma effect on quality life has been the focus of many published researches. A study done in Nigeria revealed that around a quarter of the children attending asthma clinic were psychologically impaired and asthma interfered greatly with their daily activities [4]. Furthermore, literature review on psychosocial aspects of asthma in the Arab world revealed that asthma had a significant adverse effect on the quality of life of children as indicated by the high prevalence of behavioral and emotional difficulties among them, in addition to increased frequency of school absenteeism and deteriorating academic performance [5].

Researchers also discovered a correlation between behavioral disorders and the degree of asthma control and quality of life. For example, poor asthma control was associated with clinically significant levels of behavioral problems, as reported in a study done in the United States [6]. Furthermore, Alvim et al. [7] reported that the prevalence of emotional and behavioral disorders in asthmatic adolescents was high (20.6%) in comparison to nonasthmatic adolescents (9%). According to a recent study, it was found that anxiety and depression are prevalent in those of poor asthma control [8] and behavioral disturbances were evident more often among children with severe asthma [9].

This study was conducted in Jeddah, which is considered to be the second largest city in Saudi Arabia. It is a metropolitan area on the coast of the Red Sea with a tropical climate. It has been observed that sudden climate changes and dust storms in Jeddah tend to have their effects on asthmatic children, as geographical variations were noticed to play a role in the control of asthma [10]. Also, it is worth noting that only a few researches in Saudi Arabia studied the effect of asthma on the social, behavioral, and psychological well-being of the children [11, 12].

Therefore, this study is aiming to assess the level of asthma control and its association to the quality of life of children with asthma.

## 2. Material and Methods

### 2.1. Study Design and Population

This cross-sectional study was conducted over a period of eight months. The study included 106 children with bronchial asthma who were accompanied by their caregiver to the outpatient department of King Abdulaziz University Hospital and Maternity and Children Hospital, Jeddah, Saudi Arabia. An ethical approval was received for this study from the ethical committee of both hospitals.

### 2.2. Subjects and Sampling

A convenient sampling method was adopted. Children with asthma within the age group of 7-17 years were recruited from pulmonology outpatient clinics of two hospitals in Jeddah within the period from January 2017 to June 2017. On average, there are between 10 and 15 patients in each clinic with various pulmonary diseases including asthma. Patients are usually first seen by general pediatrics before being referred to pulmonology. Consent of the caregiver and an assent of the child were provided for their participation in the study. Inclusion criteria for the study included children aged 7-17 years, with physician-diagnosed asthma, who have no positive history of any other chronic medical conditions. Patients who did not fulfill these criteria were excluded.

## 3. Tools

### 3.1. Demographics of the Sample

General demographic characteristics were collected such as age, gender, nationality, and the presence of family history of asthma. This was reported by the caregiver of the child.

### 3.2. Evaluation of Asthma Control

Validated Arabic version of the asthma control test (ACT) was used. This tool assesses general asthma symptoms and the frequency of shortness of breath, use of inhalers, and asthma influence on the child's functional status. It categorizes the children as having controlled asthma (score more than 19) or poorly controlled asthma (score that equals 19 or less) [13–15]. This was reported by both of the child and his∖her caregiver.

### 3.3. Assessment of Quality of Life

#### 3.3.1. Pediatric Asthma Quality of Life Questionnaire (PAQLQ)

A validated Arabic version of PAQLQ was used. This tool measures the functional problems (physical, emotional, and social) that are most troublesome to children as a result of their asthma. It is a self-reported questionnaire that has 23 items rated on a 7-point Likert scale from 1 (not at all) to 7 (always). Higher scores indicate better quality of life [16–18]. This was reported by the child.

#### 3.3.2. Strengths and Difficulties Questionnaire (SDQ)

A validated Arabic version of SDQ was used. It is a frequently used instrument for screening psychopathology in children and adolescents. It is a valid instrument that assesses the presence of psychosocial problems through the following domains (emotional symptoms, conduct problems, hyperactivity, peer problems, and prosocial behavior). It is comprised of 25 questions and the answers are not true, somewhat true, and certainly true. Answers were scored, respectively, in a range from 0 to 2 [19–21]. This was reported by the caregiver of the child.

### 3.4. Statistical Analysis

Data were summarized using frequencies and percentages for categorical variables or means with standard deviation (SD) for measured variables. For the univariate analysis, variables were dichotomized based on the adequacy of asthma control into (well-controlled asthma and poorly controlled asthma). Differences between continuous data were analyzed using the independent t-test, and the Chi-square or Fisher's exact tests were used to assess categorical variables as applicable. A P value ≤ 0.05 was considered significant. Statistical analysis was performed with IBM SPSS Statistical software package version 23.

## 4. Results

### 4.1. Demographics of the Sample

There were 112 eligible patients. Six of them refused to participate and there were no withdrawals during data collection. The study comprised 106 children with asthma, including 74 young children (7-12 years of age) and 32 adolescents (13-17 years of age). 75% of the sample had positive family history of asthma, and most of the sample were of Saudi nationality. Demographic characteristics and social variables of the patients and their parents are given in Table 1.

### 4.2. Evaluation of Asthma Control and Related Factors

Only 16% of the sample had controlled asthma while 84% of them had poorly controlled asthma. The status of asthma control was better among children of educated parents, employed mothers, and those having high family income (P value < 0.05). In addition, asthma duration was directly related to the adequacy of asthma control, i.e., the longer the child had asthma, the less severe it was (Table 1).

## 5. Assessment of Quality of Life and Psychosocial Well-Being

### 5.1. The Quality of Life as Assessed by PAQLQ

Significantly poorer quality of life was observed in children with uncontrolled asthma (p = <0.001) and all domains (activity, symptoms, and emotional function) were equally affected. Tables 2 and 3 provide more details.

### 5.2. Psychosocial Well-Being as Assessed by SDQ

Children with controlled and uncontrolled asthma were equally affected psychosocially with no relation between asthma control and their psychosocial well-being (p = 0.58). Also, none of the SDQ domains (the emotional, conduct, hyperactivity, peer, and prosocial problems) showed any relation with asthma control status. This is further described in Tables 2 and 3.

## 6. Discussion

Three relevant findings emerged from this study. First, asthma control status among children was surprisingly low considering that those patients were approached during a follow-up appointment. Second, the quality of life of asthmatic children was significantly lower among those with poorly controlled asthma. Lastly, there was no association between asthma control and the presence of psychosocial problems among the sample.

### 6.1. Asthma Control Status

The percentage of controlled asthma varies across the world. The reported figures in the literature are influenced by the setting of the study, sample size, and the assessment tool used.

In this study, the percentage of children with controlled asthma was 16%, which is similar to many studies worldwide, including a study done in Canada that used Canadian Pediatric Asthma Consensus Guidelines to assess asthma control among children visiting respiratory and allergy clinics, in addition to the emergency department, in which only 11% were controlled [22]. Another study done in Ta'if, Saudi Arabia, reported a percentage of 12%. Children were assessed using ACT and were recruited from pediatric outpatient clinic of Ta'if Hospital and six primary healthcare centers [11]. This small number of controlled asthmatics could be explained by the observation that the two clinics in the current study were subspecialized in pulmonology and most patients presenting to these clinics are usually referred from the general pediatric clinics due to difficulty in controlling their asthma.

On the other hand, many other studies showed contrary results, including two studies done in Riyadh: one reported that 41% of the children had controlled asthma, while the other one revealed that the majority of Saudi adolescents were considered mild asthmatics [12, 23]. However, this difference might be because, in our study, the sample was recruited from the subspecialty clinic where those with severe asthma are seen. Meanwhile, those considered mild asthmatics (probably more controlled) are seen by general practitioners, general pediatricians, and family physicians.

## 7. Assessment of Quality of Life and Psychosocial Well-Being

### 7.1. Quality of Life as Assessed by PAQLQ

Poor quality of life is significantly related to impaired asthma control, as implied by the PAQLQ scores in our study. Many other studies conducted in Iran, Unites States, and Saudi Arabia have shown similar results [11, 24–26].

In addition, all the three domains of the PAQLQ (activity, symptoms, and emotional function) were found to be equally affected. The study that was held in Ta'if showed similar findings except that the activity limitations domain was found to be the most affected domain [11]. Similarly, the study done in Iran reported that males had more disturbance in the quality of life than females, mainly in the activity domain [24].

### 7.2. Psychosocial Well-Being as Assessed by SDQ

This study showed that there was no significant difference in the psychosocial well-being on both controlled and uncontrolled groups. This goes against what Hysing et al. [27] found. In their study, the SDQ reported an increased rate of emotional and behavioral problems in children with chronic illnesses including asthma. Furthermore, the presence of psychological problems among asthmatic patients has been shown to be linked to the level of disease control [28].

Our findings could be explained by the small number of controlled asthmatic children. Moreover, it is suggested that asthma as a chronic illness could be influencing the behavioral health of the children regardless of their control status. A study done by Tibosch et al. [29] found that well-controlled asthma does not necessarily rule out major psychosocial problems. The authors believe that a prospective cohort study would measure the direct relationship between quality of life and asthma control in a more accurate way.

### 7.3. Other Factors That Were Related to Asthma Control

Parents' educational level and family income were positively related to asthma control status. Finkelstein J. a. et al. [30] found that children of parents with low educational levels are associated with medication underuse. Also, other studies in the United Kingdom and Germany have discovered that severe asthma is correlated with decreasing socioeconomic status [31, 32]. Among the plausible explanations for these findings is the point that highly educated parents are more aware of the benefits of controlling their children's asthma in addition to an easier access to healthcare facilities and medications.

Moreover, it was noticed that children of employed mothers have better control of asthma; this could be because of the direct relationship between mothers' employment status and their educational level, in addition to their ability to afford the needed medications. This finding contradicts the result of a study done in the United States which reported that maternal employment increases the likelihood for children to develop an asthma episode by 12% compared to those who are unemployed [33]. Also, 61.7% of the patients with poorly controlled asthma had history of smoking exposure in their homes. This could play a role in the status of asthma control within our sample and illustrates the importance of conducting a study that focuses on factors affecting asthma control. Nevertheless, multivariate logistic regression was not performed due to the limited number of patients with well-controlled asthma.

## 8. Limitations of the Study

Authors believe that this study needs to be implemented on a larger sample size, obtained from hospitals situated in different locations in Jeddah, so it can represent the asthma control status and its relation to quality of life adequately. Moreover, an interview-based qualitative research could yield more accurate results regarding the details of asthma effect on quality of life compared to self-administrated questionnaires. Finally, conducting a study to understand the difference between the characteristics of general clinic patients and pulmonary clinic patients would help in delineating the asthma control status accurately.

## 9. Conclusion

This study highlights the association of poorly controlled asthma with a poor quality of life. It is recommended that the quality of life of children should be assessed and observed during clinic visits for a better holistic approach and effective improvement of outcome. Further researches are needed to study the risk factors leading to poor asthma control, the psychological effect of asthma, and the importance of screening for behavioral problems among asthmatic children.

## Figures and Tables

**Table 1:** Sociodemographic characteristics of the sample.

	Controlled	Uncontrolled	P value
	n = 17	n = 89	
Age	Mean: 11	Mean: 10.337	.335
	St Dev: 2.423	St Dev: 2.615	

Gender			
Male	11 (64.7%)	55 (61.8%)	0.821

Nationality			
Saudi	11 (64.7%)	64 (71.9%)	0.550

Positive family history	11 (64.7%)	68 (76.4%)	0.310

Parents' relationship			
Married	17 (100%)	81 (91%)	0.199

Father's education			0.00
Elementary	0 (0%)	0 (0%)	
Intermediate	0 (0%)	7 (8.6%)	
High school	4 (23.5%)	40 (49.4%)	
College	10 (58.8%)	34 (42%)	
Higher studies	3 (17.6%)	0 (0%)	

Mother's education			0.044
Elementary	0 (0%)	7 (8.6%)	
Intermediate	3 (17.6%)	26 (32.1%)	
High school	5 (29.4%)	28 (34.6%)	
College	8 (47.1%)	20 24.7%)	
Higher studies	1 (5.9%)	0 (0%)	

Employed mother	12 (70.6%)	33 (40.7%)	.025

Family income			0.00
Less than 5000	0 (0%)	7 (8.6%)	
5000 – 10000	1 (5.9%)	31 (38.3%)	
10000 – 15000	6 (35.3%)	33 (40.7%)	
15000-20000	7 (41.2%)	10 (12.3%)	
20000-25000	3 (17.6%)	0 (0%)	
>25000	0 (0%)	0 (0%)	

Smoking exposure at home	5 (29.4%)	50 (61.7%)	.015

Asthma duration	Mean: 76.59	Mean: 45.88	0.00
	St Dev: 37.321	St Dev: 30.388	

**Table 2:** The scores of pediatric asthma quality of life questionnaire and the strengths and difficulties questionnaire in relation to asthma control status.

	Controlled	Uncontrolled	P value
	n = 17	n = 89	
PAQLQ^*∗*^	Mean 6.095	Mean 4.325	<0.001
	St dev 0.966	St dev 1.000	

SDQ^†^ (total difficulties score)	Mean 13.177	Mean 13.82	0.58
	St dev 4.187	St dev 4.451	

^*∗*^PAQLQ: pediatric asthma quality of life questionnaire.

^†^SDQ: strengths and difficulties questionnaire.

**Table 3:** Subanalysis of pediatric asthma quality of life questionnaire domains and the strengths and difficulties questionnaire scales in relation to asthma control status.

	Controlled	Uncontrolled	P value
	n = 17	n = 89	
(i) PAQLQ^*∗*^ domains:			

Activity	Mean 7.529	Mean 5.348	0.00
	St dev 1.302	St dev 1.358	

Symptoms	Mean 6.018	Mean 4.234	0.00
	St dev 1.035	St dev 1.096	

Emotional function	Mean 6.235	Mean 4.468	0.00
	St dev 1.035	St dev 1.154	

(ii) SDQ^†^ scales:			

Emotional problems	Mean 3.529	Mean 4.011	.364
	St dev 1.772	St dev 2.037	

Conduct problems	Mean 2.588	Mean 2.629	.926
	St dev 1.938	St dev 1.612	

Hyperactivity	Mean 3.706	Mean 3.933	.691
	St dev 2.114	St dev 2.152	

Peer problems	Mean 3.353	Mean 3.247	.760
	St dev 1.169	St dev 1.325	

Prosocial	Mean 8.412	Mean 7.573	.119
	St dev 1.805	St dev 2.0499	

^*∗*^PAQLQ: pediatric asthma quality of life questionnaire.

^†^SDQ: strengths and difficulties questionnaire.

## Data Availability

The data used to support the findings of this study are available from the corresponding author upon request.

## References

[1] Masoli M., Fabian D., Holt S., Beasley R. (2004). The global burden of asthma: executive summary of the GINA Dissemination Committee Report. *Allergy: European Journal of Allergy and Clinical Immunology*.

[2] Nahhas M., Bhopal R., Anandan C., Elton R., Sheikh A. (2012). Prevalence of Allergic Disorders among Primary School-Aged Children in Madinah, Saudi Arabia: Two-Stage Cross-Sectional Survey. *PLoS ONE*.

[3] Alqahtani J. M. (2016). Asthma and other allergic diseases among Saudi schoolchildren in Najran: The need for a comprehensive intervention program. *Annals of Saudi Medicine*.

[4] Tunde-Ayinmode M. F. (2015). Children with bronchial asthma assessed for psychosocial problems in a teaching hospital in Nigeria. *African Health Sciences*.

[5] Al-khateeb A. J., Al khateeb J. M. (2015). Research on psychosocial aspects of asthma in the Arab world: a literature review. *Multidisciplinary Respiratory Medicine*.

[6] Weil C. M., Wade S. L., Bauman L. J., Lynn H., Mitchell H., Lavigne J. (1999). The relationship between psychosocial factors and asthma morbidity in inner-city children with asthma. *Pediatrics*.

[7] Alvim C. G., Ricas J., Camargos P. A., Lasmar L. M., Andrade C. R., Ibiapina C. d. (2008). Prevalência de transtornos emocionais e comportamentais em adolescentes com asma. *Jornal Brasileiro de Pneumologia*.

[8] Coban H., Aydemir Y. (2014). The relationship between allergy and asthma control, quality of life, and emotional status in patients with asthma: a cross-sectional study. Allergy. *Allergy, Asthma & Clinical Immunology*.

[9] McNichol K., Williams H., Allan J., McAndrew I. (1973). Spectrum of asthma in children. III. Psychological and social components. *British Medical Journal*.

[10] Malhotra K., Baltrus P., Zhang S., Mcroy L., Immergluck L. C., Rust G. (2014). Geographic and racial variation in asthma prevalence and emergency department use among Medicaid-enrolled children in 14 southern states. *The Journal of Asthma*.

[11] Al Zahrani S. S., El-Morsy E.-M. A., Dorgham L. S. (2014). The impact of bronchial asthma on quality of life among affected children and adolescents in Taif city, Saudi Arabia. *Life Science Journal*.

[12] Vazquez-tello A. (2016). Impact of Asthma on the Quality of Life of Adolescent Patients from Saudi Arabia. *Journal of Lung Diseases & Treatment*.

[13] Hallit S., Raherison C., Waked M., Salameh P. (2017). Validation of asthma control questionnaire and risk factors affecting uncontrolled asthma among the Lebanese children's population. *Respiratory Medicine*.

[14] El Mouzan M. I., Al Salloum A. A., Al Herbish A. S., Al Omar A. A., Qurachi M. M. (2003). Does consanguinity increase the risk of bronchial asthma in children?. *Asthma Control Test*.

[15] Nathan R. A., Sorkness C. A., Kosinski M., Schatz M. (2004). Development of the asthma control test: a survey for assessing asthma control. *Journal of Allergy and Clinical Immunology*.

[16] Juniper E. F., Guyatt G. H., Epstein R. S., Ferrie P. J., Jaeschke R., Hiller T. K. (1992). Evaluation of impairment of health related quality of life in asthma: Development of a questionnaire for use in clinical trials. *Thorax*.

[17] Abdel Hai R., Taher E., Abdel Fattah M. (2010). Assessing validity of the adapted Arabic Paediatric Asthma Quality of Life Questionnaire among Egyptian children with asthma. *Eastern Mediterranean Health Journal*.

[18] Lahaye M., Broeck N. V., Bodart E., Luminet O. (2013). Predicting quality of life in pediatric asthma: the role of emotional competence and personality. *Luminet*.

[19] He J.-P., Burstein M., Schmitz A., Merikangas K. R. (2013). The strengths and difficulties questionnaire (SDQ): The factor structure and scale validation in U.S. Adolescents. *Journal of Abnormal Child Psychology*.

[20] Goodman R. (1997). The strengths and difficulties questionnaire: a research note. *Journal of Child Psychology and Psychiatry and Allied Disciplines*.

[21] Alyahri A., Goodman R. (2006). Validation of the Arabic strengths and difficulties questionnaire and the development and well-being assessment. *Eastern Mediterranean Health Journal*.

[22] Cope S. F., Ungar W. J., Glazier R. H. (2008). Socioeconomic factors and asthma control in children. *Pediatric Pulmonology*.

[23] BinSaeed A. A., Torchyan A. A., Alsadhan A. A. (2014). Determinants of asthma control among children in Saudi Arabia. *Journal of Asthma & Allergy Educators*.

[24] Zandieh F., Moin M., Movahedi M. (2006). Assessment of quality of life in Iranian asthmatic children, young adults and their caregivers. *Iranian Journal of Allergy, Asthma and Immunology*.

[25] Schmier J. K., Manjunath R., Halpern M. T., Jones M. L., Thompson K., Diette G. B. (2007). The impact of inadequately controlled asthma in urban children on quality of life and productivity. *Annals of Allergy, Asthma & Immunology*.

[26] Levy J. I., Welker-Hood L. K., Clougherty J. E., Dodson R. E., Steinbach S., Hynes H. P. (2004). Lung function, asthma symptoms, and quality of life for children in public housing in Boston: A case-series analysis. *Environmental Health: A Global Access Science Source*.

[27] Hysing M., Elgen I., Gillberg C., Lundervold A. J. (2009). Emotional and behavioural problems in subgroups of children with chronic illness: Results from a large-scale population study. *Child: Care, Health and Development*.

[28] Baiardini I., Sicuro F., Balbi F., Canonica G. W., Braido F. (2015). Psychological aspects in asthma: do psychological factors affect asthma management?. *Asthma Research and Practice*.

[29] Tibosch M., Reidsma C., Landstra A. (2010). An asthma-related quality of life instrument is unable to identify asthmatic children with major psychosocial problems. *European Journal of Pediatrics*.

[30] Finkelstein J. A., Lozano P., Farber H. J., Miroshnik I., Lieu T. A. (2002). Underuse of controller medications among medicaid-insured children with asthma. *JAMA Pediatrics*.

[31] Mielck A., Reitmeir P., Wjst M. (1996). Severity of childhood asthma by socioeconomic status. *International Journal of Epidemiology*.

[32] Duran-Tauleria E., Rona R. J. (1999). Geographical and socioeconomic variation in the prevalence of asthma symptoms in English and Scottish children. *Thorax*.

[33] Morrill M. S. (2011). The effects of maternal employment on the health of school-age children. *Journal of Health Economics*.

